# *In situ-*Like Aerosol Inhalation Exposure for Cytotoxicity Assessment Using *Airway-on-Chips* Platforms

**DOI:** 10.3389/fbioe.2020.00091

**Published:** 2020-02-20

**Authors:** Shani Elias-Kirma, Arbel Artzy-Schnirman, Prashant Das, Metar Heller-Algazi, Netanel Korin, Josué Sznitman

**Affiliations:** Department of Biomedical Engineering, Technion – Israel Institute of Technology, Haifa, Israel

**Keywords:** pulmonary, airway, organ-on-chip, *in vitro*, human primary cells, inhalation, aerosol, cytotoxicity

## Abstract

Lung exposure to inhaled particulate matter (PM) is known to injure the airway epithelium via inflammation, a phenomenon linked to increased levels of global morbidity and mortality. To evaluate physiological outcomes following PM exposure and concurrently circumvent the use of animal experiments, *in vitro* approaches have typically relied on traditional assays with plates or well inserts. Yet, these manifest drawbacks including the inability to capture physiological inhalation conditions and aerosol deposition characteristics relative to *in vivo* human conditions. Here, we present a novel *airway-on-chip* exposure platform that emulates the epithelium of human bronchial airways with critical cellular barrier functions at an air–liquid interface (ALI). As a proof-of-concept for *in vitro* lung cytotoxicity testing, we recapitulate a well-characterized cell apoptosis pathway, induced through exposure to 2 μm airborne particles coated with αVR1 antibody that leads to significant loss in cell viability across the recapitulated airway epithelium. Notably, our *in vitro* inhalation assays enable simultaneous aerosol exposure across multiple airway chips integrated within a larger bronchial airway tree model, under physiological respiratory airflow conditions. Our findings underscore *in situ-*like aerosol deposition outcomes where patterns depend on respiratory flows across the airway tree geometry and gravitational orientation, as corroborated by concurrent numerical simulations. Our *airway-on-chips* not only highlight the prospect of realistic *in vitro* exposure assays in recapitulating characteristic local *in vivo* deposition outcomes, such platforms open opportunities toward advanced *in vitro* exposure assays for preclinical cytotoxicity and drug screening applications.

## Introduction

With a vast and highly vascularized surface optimized for gas exchange (Grotberg, 2011; Janssen et al., 2016), the lungs constitute the largest organ directly exposed to the external environment. As such, the lungs are vulnerable to a breadth of threats associated with occupational (Leggat et al., 2007) and environmental (Brulle and Pellow, 2006) hazards. In particular, exposure to inhaled airborne particulate matter (PM) represents a global health problem due to adverse cardiovascular and respiratory outcomes linked to increased morbidity and mortality (Anderson et al., 2012; Kim et al., 2015; Conibear et al., 2018; Wu et al., 2018). To protect the body from continuous exposure to foreign PM, and also pathogens and other toxic chemicals (Bals and Hiemstra, 2004), the lungs’ luminal surface is populated with a confluent, uninterrupted epithelial cell carpet that exists as a continuum across the airway tree (Daniels and Orgeig, 2003). In the conducting airways specifically, epithelial cells are covered with an extracellular periciliary lining (Button et al., 2012) itself immersed under a mucus layer present at an air–liquid interface (ALI) (Fahy and Dickey, 2010). Briefly, the secretion of mucus by goblet and club cells (Barnes et al., 2003; Lai et al., 2008; Fahy and Dickey, 2010; Whitsett, 2018), in combination with ciliated cells, contributes to airway clearance (Janssen et al., 2016) by trapping (Stannard and O’Callaghan, 2006) inhaled particles and pathogens (e.g., bacteria) depositing on the pulmonary epithelium. This complex cellular environment requires a well-differentiated bronchial epithelium that constitutes the conducting airways’ innate defense mechanisms and maintains pulmonary homeostasis.

In an effort to evaluate physiological outcomes following PM exposure and its association with diseases (e.g., COPD; Anderson et al., 2012), *in vivo* animal experiments have been traditionally pursued. For example, *in vivo* studies have demonstrated that pulmonary exposure to PM causes lung inflammation and oxidative stress (Inoue et al., 2006; Nemmar et al., 2009, 2011). Nemmar et al. (2009) showed that 24 h post intratracheal instillation of diesel exhaust particles, the influx of macrophages and neutrophils in broncho-alveolar mice lavages was elevated. Despite such progress, *in vivo* animal studies remain contentious due to critical differences in anatomy, immune systems, and inflammatory responses compared with humans (Benam et al., 2015), thereby raising the need for more relevant platforms for evaluation (Bueters et al., 2013). To overcome such drawbacks and uncover cellular mechanisms in which PM toxicity affects the respiratory system, *in vitro* studies with cell cultures have been actively sought (Paur et al., 2011; Nemmar et al., 2013; Drasler et al., 2017). In particular, ALI conditions can be recapitulated by culturing cells on the apical side of a porous membrane using for instance commercially available Transwell inserts; such setups are indeed critical in striving to mimic physiologically relevant characteristics of the bronchial airway lumen, including for example mucus secretion (Grainger et al., 2006; Lin et al., 2007). Furthermore, capturing biological airway responses specific to humans following PM exposure to toxins (Mustafiz et al., 2015) calls for the use of human primary cells (Skardal et al., 2016) in an effort to overcome ongoing limitations with animal studies. Although the aforementioned macroscopic *in vitro* approaches reproduce some of the cellular functions of the human pulmonary environment, both true-scale anatomical airway features and physiological (air) flow conditions are still widely absent from existing *in vitro* exposure assays. Such drawbacks continue to restrict *in vitro* setups from addressing faithfully the physical aerosol transport determinants leading to PM deposition along the inhalation route. Since direct *in vivo* human exposure studies are ethically controversial, the development of realistic *in vitro* human exposure assays is thus desired to advance our understanding of inflammation and disease following harmful PM exposure under realistic *in situ* inhalation conditions.

Over the past decade, *organ-on-chips* have gained momentum in laying the foundations for constructing attractive *in vitro* models that mimic more realistically physiologically relevant organ functions in humans (Nesmith et al., 2014; Abaci et al., 2015; Bovard et al., 2018; Ronaldson-Bouchard and Vunjak-Novakovic, 2018; Shirure et al., 2018). Such platforms allow *in vitro* examinations within micro-devices lined with human cells, thereby fostering new physiological insights, in both health and disease, complementary to current tools available for diagnostics and conventional *in vitro* approaches (Tenenbaum-Katan et al., 2018). Specifically, lung-related models have been devised to mimic the human alveolar–capillary interface and stimulate inflammatory responses *in vitro* (Huh et al., 2010, 2012; Artzy-Schnirman et al., 2019b). For example, Huh et al. (2010) demonstrated the importance of using cyclic mechanical strain, which accentuates toxic and lung inflammatory responses to silica nanoparticles, associated with the development of vascular leakage, which leads to pulmonary edema. In parallel, Benam et al. (2015) reconstituted a human lung *small airway-on-a-chip* model by co-culturing both endothelium cells and epithelial tissue from healthy individuals and COPD patients to model human lung inflammatory disorders. In addition, using the *small airway-on-a-chip* model connected to a smoking instrument, Benam et al. (2016) established a platform to study a patient-specific response to inhaled smoke. Punde et al. (2015) showed protein-induced lung inflammation and emphasized the need of using flow, which allows reconstituting the blood vessel–tissue interface for *in vitro* assays, and by that improve pre-clinical studies. Together with other recent efforts (Douville et al., 2011; Nesmith et al., 2014; Stucki et al., 2014; Li et al., 2015; Hassell et al., 2017; Humayun et al., 2018), these studies have brought tremendous progress in lung research but are still widely limited to platforms with a single airway channel, thus short of mimicking the complexity of the conducting airway tree. This is especially critical when exploring the fate of inhaled aerosols where the coupling between respiratory airflows and lung anatomy is known to modulate particle deposition outcomes (Kleinstreuer and Zhang, 2010; Fishler et al., 2015; Koullapis P.G. et al., 2018).

Motivated by the critical need to deliver *in vitro* platforms that mimic more closely physiological inhalation conditions of human lung exposure with characteristic *in situ*-like deposition outcomes (Artzy-schnirman et al., 2019a), we have established a novel inhalation assay integrated with an *airway-on-chip* platform comprising three-generational bronchial airways cultured with a differentiated human bronchial epithelium. Specifically, such *in vitro* inhalation assay reproduces mechanistically faithful aerosol transport determinants leading to airway deposition at a functional epithelial barrier. As a proof-of-concept, we characterize PM toxicity whereby we recapitulate a well-characterized apoptosis pathway (Agopyan et al., 2003, 2004). One of the better-known pathways in which PM affects human airway cytotoxicity is mediated by receptors which induce cell apoptosis (Becker et al., 1996; Holian et al., 1998), including importantly the Vanilloid (i.e., VR1) receptor (Agopyan et al., 2003). Seminal *in vitro* studies using primary cells have shown that 48 h post PM particle stimulation the level of apoptotic cells is elevated by activation of such VR1 receptor (Agopyan et al., 2004). Here, apoptosis is induced through exposure to 2-μm airborne particles coated by αVR1 antibody; an aerosol size known to deposit in the small bronchi and bronchioles (Bair, 1989; Stahlhofen et al., 1989; Hinds, 1999). Importantly, our *in vitro* inhalation assays deliver aerosols simultaneously across four *airway-on-chips* integrated within a larger conductive airway tree model to explore *in situ-*like aerosol deposition outcomes under physiological respiratory airflows and for various gravitational orientations. Given that current cytotoxicity assays are still widely based on either non-physiological liquid installations of PM suspensions (Hittinger et al., 2017) or alternatively direct spraying at an ALI (Blank et al., 2006; Lenz et al., 2014; Röhm et al., 2017), our *airway-on-chip* exposure setup is part of steadfast efforts (Artzy-schnirman et al., 2019a) leading toward a new generation of advanced *in vitro* lung toolkits for human inhalation toxicity assays.

## Materials and Methods

### Airway-on-Chip

#### Device Design

**FIGURE 1:**
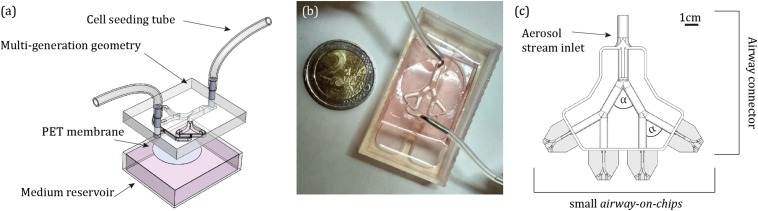
Design of the *airway-on-chip* platform and its integration for aerosol inhalation exposure assays. **(a)** Computer-aided drawing (CAD) schematic of the device including the airway tree model (apical side), a porous PET membrane (gray circle) separating the bottom reservoir for medium (basal side). **(b)** Photograph of the assembled device. **(c)** CAD schematic of the custom-designed 3D airway tree connector which allows to simultaneously perform experiments across four individual *airway-on-chip* devices (shown in gray), and control the orientation of gravity during an exposure assay, with bifurcating angle of α = 60° (Supplementary Figure S1).

The design of the proposed *airway-on-chips* consists of a planar, symmetric airway tree spanning three generations (with a total of four outlets), as schematically shown in Figure 1a. Individual airways consist of lumens of semi-circular cross-sections (chosen as a result of the need for a porous membrane for cell culture, see below) where dimensions, summarized in Table 1, are based on morphometric measurements representative of a typical human adult lung, following the seminal works of Weibel et al. (1963) and Horsfield et al. (1971). Note that our *in vitro* platform broadly mimics small bronchial branches of the conducting zone, with airway diameters <2.5 mm, along with an idealized constant planar bifurcating angle of 60° across all generations (Ménache et al., 2008). In line with recent *lung-on-chip* designs limited to single airway channels, the present design allows to culture cells at ALI conditions together with access for inhalation airflows through the apical compartment using a syringe pump (see below).

**TABLE 1:** Dimensions of the *airway-on-chip* platform.

**Generation**	**Length (mm)**	**Diameter (mm)**
1	6.59	2.20
2	5.55	1.68
3	3.58	1.25

#### Device Fabrication

The model negative is 3D printed in house via stereo-lithography (Formlabs, Form 2). The mould is used as a master template for polydimethylsiloxane (PDMS) casting. PDMS was mixed with a curing agent (Dow Corning) at a 10:1 weight ratio, poured on the template and baked for 4 h at ∼65°C. Cured PDMS was subsequently pealed from the mould and punched using a biopsy punch of 1 mm size (Miltex, 3331) to create inlet and outlets. Next, a 10 μm thick polyethylene terephthalate (PET) membrane with 0.4 μm pore size (Corning, CLS3450) was bonded to the PDMS model and channels were irreversibly sealed. Upon seeding cells on the apical side of the PET membrane, the model is placed above a medium reservoir (i.e., basal side). The completed assembly of the platform is presented in Figure 1b.

### Aerosol Exposure Experiments

#### Particle Preparation

Following an established protocol, 2 μm monodispersed polystyrene (PS) particles [FluoSpheres Carboxylate-Modified microspheres, Red Fluorescent (580/605), 2% solid F8826, Thermo Scientific] were coupled to αVR1 antibody (Baker et al., 2019) (Abcam, ab3487)/bovine serum albumin (BSA; MP Biomedicals, 160069) in phosphate-buffered saline (PBS; Sigma-Aldrich, D8537), respectively, using a two-step *EDC*/*Sulfo NHS Covalent Coupling Procedure* (Merck, 2015) (Merck Millipore). Such protocol yields 2 μm monodispersed PS particles conjugated with αVR1 antibody/BSA. Briefly, after 15 min sonication, 250 μl PS particles were suspended in 250 μl of 50 mM MES buffer solution (pH 6). The suspension was vortexed and centrifuged at 3000 × *g* for 5 min at 4°C. This washing procedure was repeated three times. Next, 12 μl of 200 mM EDC reagent solution and 120 μl of 200 mM Sulfo-NHS reagent solution were added to the particle suspension with 250 μl of MES buffer. The suspension was vortexed and mixed gently in a vertically rotating plate for 30 min at room temperature (RT), followed by three washes with 1 ml MES buffer. Next, 33 μl of αVR1 antibody (1 mg/ml)/100 μl of BSA (1 mg/ml), respectively, were added to the particles with 1 ml MES buffer, vortexed and mixed gently in a vertically rotating plate for 3 h at RT. The suspension was washed from the unreacted protein using a spin down centrifugation cycle (the supernatant was kept for protein content analysis), and the particles were suspended in 1 ml MES buffer and 15 μl of ethanolamine to quench the reaction. Finally, the particles were washed three times using 1 ml blocking buffer [containing 0.5% (w/v) casein in MES buffer], and three times with 1 ml of PBS. The particles were kept at 4°C until used for an experiment. To assess qualitatively the PS-VR1 conjugation, particles were incubated for 1 h at RT with secondary antibody Alexa Fluor 488 anti-rabbit, before confocal microscopy imaging of fluorescent immunostaining was performed on the conjugated particles (Supplementary Figure S2).

#### Aerosol Exposure Assay

The present *in vitro* aerosol exposure assays strive to recapitulate *in situ*-like inhalation conditions. In a first step, aerosol exposure assays were conducted in the absence of cells to characterize *in vitro* aerosol transport determinants and examine solely aerosol deposition patterns inside the devices. Subsequently, the identical protocol was implemented to explore cytotoxicity on the epithelium. Briefly, a monodispersed aerosol was produced by aerosolization (Fishler et al., 2015) of PBS-suspended 2 μm red fluorescent PS microspheres conjugated to αVR1 antibody (as described above) using an aerosol generator (TSI, 3076) with air as the gas source and subsequently drying the PBS droplets using two consecutive diffusion driers (TSI, 3062). The rationale for selecting such particle size follows as 2 μm represents a good candidate for aerosol deposition in the deep tracheobronchial (TB) regions (Bair, 1989; Stahlhofen et al., 1989; Hinds, 1999). To avoid aggregate formation prior to the deposition assays, the microspheres were first sonicated in a water bath for 20 min. Next, the 2 μm PS particles were diluted in distilled water to a concentration of 1 × 10^7^ particle/ml for aerosol exposure, while for the cell viability assay in the cultured *airway-on-chips*, particles were diluted in PBS to a concentration of 4.55 × 10^8^ particle/ml (see the section “Results”). The aerosol flow was fed through an antistatic tube where the flow rate was controlled by a pinch valve. To deliver a physiologically relevant exposure assay, the flow rate was further reduced by splitting the main aerosol stream into four paths integrated within a custom-designed conducting 3D airway tree model that approximately mimics mid-bronchial generations in an average adult human lung (Ménache et al., 2008; Figure 1c and Supplementary Figure S1).

Using the 3D airway connecting model enables to simultaneously perform *n* = 4 *airway-on-chip* experiments, directly integrated within an anatomically inspired conducting airway tree. The overall flow rate selected was 0.2 l/min, as measured using a flow meter (TSI, 4100) at the aerosol generator outlet; the ensuing flow rate at the entrance of each (four) device was 0.05 l/min, i.e., corresponding to physiological airflow conditions representative of flow phenomena in small bronchial airways. Specifically, we replicate quiet breathing conditions (Pedley, 2003; Sznitman, 2013), such that the characteristic Reynolds numbers (Re) range between approximately 22 and 10 across the three generations of the *airway-on-chip*, where Re = *ud*/ν and represents the relative magnitude of the inertial forces to viscous forces (i.e., ν is the kinematic viscosity of air, *u* is the characteristic mean airflow velocity in each generation of diameter *d*).

In each experiment, four *airway-on-chip* devices are positioned in a horizontal orientation relative to gravity and directly tight-fitted to the four outlets of the 3D airway tree connector along the streamwise flow direction (see Figure 1c and Supplementary Figure S1). Unlike cell seeding via the top of the device (Figures 1a,b), this design ensures anatomical continuity and thereby adequate respiratory airflows during aerosol exposure assays. Note that the influence of gravity, and thus particle deposition, can be modified with the present design by changing the device position relative to the connector’s outlet (Supplementary Figure S3). Each exposure assay was conducted for 30 min in an effort to yield representative ensemble depositions of aerosols and in parallel compare these with numerical simulation (see the section “Results”); in the case of cell viability assays a continuous exposure was conducted for 5 min. Note that here, constant steady-state inhalation flow conditions are mimicked, giving rise to fully developed laminar flow conditions; the range of Re across the 3D airway connector ranges approximately between Re ∼ 50–22, in line with physiological respiratory conditions (Pedley, 2003; Sznitman, 2013).

To quantify ensuing aerosol deposition patterns, the models were subsequently imaged under microscopy using an inverted fluorescent microscope (Nikon eclipse Ti) at 20× magnification. A resulting single, high-resolution image of the complete *airway-on-chips* was obtained by tiling multiple images where the location of each deposited fluorescent particle was identified by locating local intensity maxima (ImageJ).

### Numerical Simulations

#### Computational Fluid Dynamics Simulations

In an effort to interpret the underlying physical aerosol transport determinants governing deposition outcomes and further compare the experimentally obtained particle deposition patterns, *in silico* flow simulations were carried out using a commercial software (Fluent 18.2, ANSYS Inc.) that solves the mass and momentum (i.e., Navier–Stokes) equations numerically. An airway model identical to the *airway-on-chip* design was first discretized with tetrahedral cell elements using the original CAD model and subsequently refined and converted into a polyhedral mesh (Inthavong et al., 2019). Flow convergence tests were conducted on three different mesh sizes, ranging between 240,000 and 660,000 polyhedral cells in size. A final mesh consisting of approximately 540,000 polyhedral cells was selected for flow simulations. To accurately mimic experimental conditions, a steady-state, fully developed velocity profile was imposed at the model inlet. A no-slip boundary condition was implemented at the walls, and any particles impacting the walls were assumed to be deposited. All four outlets were set to identical, zero-pressure outlet conditions. Given the relatively low-Reynolds-number regime (Re << 100), a laminar solver was chosen to simulate airflows. A SIMPLE pressure–velocity coupling scheme with least squares cell-based gradient discretization, second-order for pressure and second-order upwind scheme for momentum was implemented.

#### Particle Deposition Simulations

We simulated ∼6,400 monodisperse 2 μm diameter PS particles (ρ_*p*_ = 1.05 g/cm^3^), tracked using a Lagrangian one-way coupled, steady-state discrete phase model. As particle mass flow through a cross-section may be assumed proportional to flow rate, particles were seeded at the inlet with a quasi-parabolic initial distribution using a truncated normal probability density function. For such particle size, the main forces governing transport are viscous drag (i.e., low-Re Stokes drag) and gravitational sedimentation; that is Brownian motion, electrostatic charge, or other forces are neglected (Koullapis et al., 2016). Locations of the deposited particles on the bottom wall (analogous to the apical side of the PET membrane) were extracted and identified according to airway branch generation.

### Cell Culture

#### Cell Culture Maintenance

In the footsteps of recent small *airway-on-chip* models led by Benam et al. (2015), Normal Human Bronchial Epithelial (NHBE) cells (Lonza, CC-2540S) were cultured under immersed conditions with B-ALI growth medium (Lonza, 00193516) and 50% of supplemented BEGM SingleQuots (Lonza, CC-4175) in a tissue culture T75-flask (TPP, 90025). The medium was changed every second or third day until cells reached ∼90% confluency. A Trypsin–EDTA solution (Lonza, CC-5034) was then used to detach cells from culture dishes. Thereafter, cells were used for experiments as described in detail below. Cells were incubated at 37°C in a humidified atmosphere containing 5% CO_2_. Cells were cultured up to passage (Rayner et al., 2019) four; mycoplasma controls were performed routinely using MycoAlert mycoplasma detection kit (Lonza, BELT07-218) without exhibiting infection.

#### Air–Liquid Interface (ALI)

For *airway-on-chip* cultures, 1.8 × 10^5^ NHBE cells (in 135 μl medium) were seeded on top of the apical side of a 0.03 mg/ml collagen type I coated (Corning, 354236) PET membrane inside the devices, and 1 ml of medium was added to the basal compartment. For Transwell inserts culture (Corning, CLS3470), 5 × 10^4^ NHBE cells (in 200 μl medium) were seeded on a collagen type I-coated PET membrane inside inserts, and 500 μl of medium was added to the basal compartment. Both cell cultures on chip and on inserts were first conducted under immersed conditions with B-ALI growth complete medium and 1% Antibiotic Antimycotic Solution (Sigma-Aldrich, A5955). By day 4 the NHBE culture was exposed to air (i.e., ALI) by removing the medium from the apical side of the *airway-on-chip* devices and the inserts, respectively. B-ALI differentiation medium (StemCell, 05001) was introduced in the basal chamber of the devices and inserts, respectively. Differentiated NHBE cell cultures were imaged under microscopy starting from day 21 after exposure to ALI.

### Imaging

#### Scanning Electron Microscopy

Samples were washed three times with PBS and fixed in primary fixative buffer [1% para formaldehyde (PFA) and 2% GA in 0.1 M NaP pH 7.4 and 3% sucrose] for 60 min at RT. Following three washes with 0.1 M cacodylate buffer (pH 7.4) samples were fixed with 1% Osmium tetroxide in 0.1 M cacodylate buffer for 15 min at RT. Next, samples were dehydrated through a graded ethanol series and further processed by critical point drying and sputter coating with chromium (5 nm). Images were acquired with a Zeiss ULTRA plus field emission scanning electron microscope (SEM).

#### Mucus Visualization

Following perfusion of PBS, NHBE cells were fixed within the devices and inserts using 4% PFA (Sigma–Aldrich, 47608) for 20 min at RT and were washed again three times with PBS. For detection of glycoproteins typically present in mucus (Leonard et al., 2010; Lechanteur et al., 2018), samples were treated with 10 mg/ml alcian Blue 8G× (Sigma–Aldrich, A5268) in 3% acetic acid (1% w/v) for 15 min at RT and were washed three times with PBS. Images were then acquired with a light inverted microscope (Nikon Eclipse TS100) at 10×.

#### Immunofluorescence Microscopy

Directly after cell fixation using 4% PFA, cells were treated with 0.05% Triton X-100 (Sigma–Aldrich, T8787) for 3 min at RT to increase membrane permeability and were blocked for non-specific binding using 2% BSA for 1 h at RT. For F-actin staining, NHBE cells were incubated with Alexa Fluor 568 Phalloidin (ThermoFisher Scientific, A12380) diluted with PBS (ratio of 1:200) for 40 min at RT. For DAPI nucleic acid staining cells were incubated with DAPI solution (ThermoFisher Scientific, D1306), diluted with PBS (ratio of 1:400) for 5 min at RT. For tight junction proteins, Zonula occludens-1 (ZO1), cells were incubated with the primary antibodies rabbit anti-ZO1 (ThermoFisher Scientific, 617300) diluted with PBS (ratio of 1:200) overnight at 4°C, followed by incubation with secondary antibody Alexa Fluor 488 anti-rabbit (Jackson ImmunoResearch, 111-545-144), diluted with PBS (ratio of 1:400) for 1 h at RT. After each step, cells were washed three times with PBS. Finally, confocal microscopy imaging of fluorescent immunostaining was performed (Nikon Eclipse Ti with spinning disk, Yokogawa, Japan).

#### Apoptosis Quantification

Following 5–8 days under immersed conditions, NHBE cells in the devices were exposed for 5 min to the PS-αVR1 particles and then incubated for 48 h. To ensure that apoptosis resulted solely from the exposure assay, the response of two control groups was monitored: (i) NHBE cells treated with airflow and (ii) cells exposed to PS-BSA particles (without the αVR1 antibody). To quantify apoptosis, following 48 h of incubation, cells were fully harvested using a Trypsin–EDTA solution, collected and then incubated with Alexa Fluor 488 conjugated Annexin V (Abcam, ab14085) in the dark for 5 min at RT. The cells were then imaged using an imaging flow cytometer (Amnis, ImageStreamX Mark II) at 40×. As a positive control, the NHBE cells’ apoptotic response was measured over time following exposure to αVR1 antibody. Briefly, 5 × 10^4^ NHBE cells per well were seeded in a 96-well plate (Nunc, 167008). Two days after seeding, medium containing 0.66 μM αVR1 antibody was added, followed by incubation of 24 and 48 h. Next, apoptotic cells were stained using Annexin V (see details above), and imaging of fluorescent immunostaining was performed. In parallel, as an additional positive control, we checked the toxicity effects of PS-VR1 particles using NHBE cells which were seeded on top of an insert for 7 days under immersed conditions, followed by incubation of 48 h with PS-BSA and PS-VR1 in a particle:cell ratio of 1:50. Finally, confocal imaging of fluorescent immunostaining was performed.

### Particles Toxicity Assays

To ensure that apoptosis was due to the exposure assay using αVR1 antibody, a toxicity test was first performed. Briefly, 5 × 10^4^ NHBE cells per well were seeded in a 96-well plate; 2 days following seeding, medium containing 2 μm PS-BSA particles with particle:cell ratios of 5:1, 2.5:1, 1:1, 1:2.5, 1:5, 1:10, 1:100, and 1:200 were added, respectively. NHBE cells were returned for 48 h incubation. Next, a cell viability reagent (almarBlue; Bio-Rad, BUF012A) with 10% volume in culture medium was added to each well followed by 4 h incubation. Finally, absorbance measurements were performed using a plate reader (Varioskan LUX, ThermoFisher Scientific) at 570 and 600 nm wavelengths, respectively. Each test was repeated independently three times. As a positive control, medium containing 2 μm PS-VR1 particles with a particle:cell ratio of 1:100 was added in the same experiment and a viability assay using almarBlue was performed.

### Statistical Analysis

A student’s *t*-test (two-tailed) was used to determine the level of significance among different experiments device. Error bars are presented as the standard error and asterisks indicating significance in the figures correspond to *p* < 0.05, 0.005, and 0.001 shown as ^∗^, ^∗∗^, and ^∗∗∗^, respectively.

## Results and Discussion

To emulate a small conducting airway environment in view of cytotoxicity exposure evaluations, we designed a tree-like device featuring two planar, symmetric branching airway channels, broadly resembling the anatomy of small bronchial airways of an average adult lung (Figures 1a,b). The device consists of three layers (see the section “Materials and Methods”), including the apical side of the PDMS airway tree, in which the two small punches (1 mm in diameter) first allow to seed the pulmonary cells on top of the PET membrane (10 μm thick with 0.4 μm pores size). The PET membrane is bonded to the bottom PDMS compartment and sealed above by the airway channels. This permeable membrane allows nutrient supply from the medium in the reservoir and prevents cell leakage to the basal side. Notably, our custom design allows to culture the seeded pulmonary cells at ALI conditions; a necessary condition for adequate differentiation (Grainger et al., 2006; Lin et al., 2007). Our design mimics the *in vivo* environment by combining a 3D true scale pulmonary tree geometry with realistic airflow conditions and recapitulating *in situ*-like aerosol inhalation exposure to the deep TB regions of the lungs.

### Aerosol Inhalation Exposure Assay

We first explore deposition patterns of inhaled aerosols during the inhalation maneuver and present ensemble deposition patterns for 2 μm particles on the bottom surface of the *airway-on-chips* (i.e., PET membrane). Such results follow a steady (i.e., constant flow rate) inhalation aerosol exposure assay across the anatomically realistic 3D airway connector (Supplementary Figure S1). Devices were exposed for 30 min to a steady stream of aerosols at a controlled flow rate matching physiological airflow conditions representative of small bronchial (with an entrance flow rate of *Q* = 0.05 l/min). We recall that our inhalation assays omit the oscillatory nature of respiratory flows and instead focus on inhalation phenomena. We recently explored in *in silico* simulations particle deposition occurring during inhalation and exhalation phases across extensive deep lung models (Koullapis P.G. et al., 2018) and assessed the contribution of each phase, respectively. Here, rather, the focus of the present proof-of-concept lies in recapitulating for the first time real mechanistic deposition determinants of inhaled aerosols within *lung-on-chip* platforms.

**FIGURE 2:**
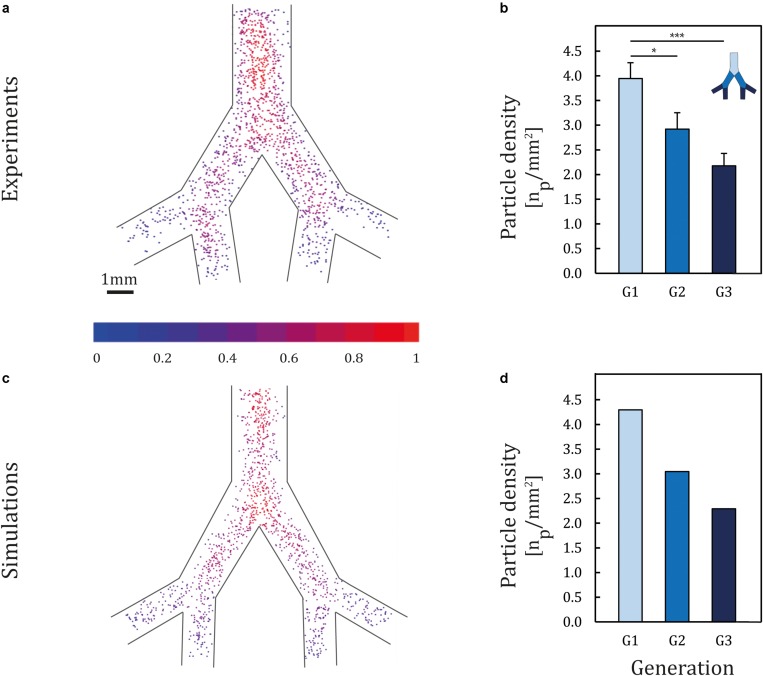
Particle deposition patterns following exposure. Two micrometers of PS particles were aerosolized into the *airway-on-chips* in a horizontal position with respect to gravity at physiological airflow conditions corresponding to quiet breathing. **(a)** Ensemble deposition patterns imaged under fluorescence microscopy (*n* = 8 models). The color-coded heat map quantifies particle concentration, defined as the number of neighboring particles within an 0.5 mm radius (results are normalized by the highest concentration in the tree). **(b)** Histogram of particle deposition density quantifying the average particle number in each generation normalized by airway area (mm^2^). Error bars represent the standard error. Asterisks indicate significance corresponding to *p* < 0.05, 0.001 shown as * and ***, respectively. Corresponding **(c)** aerosol deposition patterns and **(d)** histograms of deposition density obtained from *in silico* CFD simulations.

To mimic the hydrophilic environment of the bronchial lumen surrounded by mucus, we used collagen-coated devices (0.03 mg/ml collagen type I); we recall that in view of quantifying solely mechanistic aerosol transport characteristics devices are first void of epithelium. Figure 2A presents ensemble results of particle deposition patterns inside the *airway-on-chips* under physiological airflow conditions; the color-coded heat map quantifies particle concentration, defined as the number of neighboring particles within a 0.5 mm radius (results are normalized by the highest concentration in the tree). We begin by observing a heterogeneous deposition pattern with high particle concentrations along the centerline of each generation compared to the peripheral areas (i.e., nearer the airway side walls), in line with Poiseuille velocity profiles characteristics at such Re. When examining the average particle density (defined as the average number of deposited particles in each branch, normalized by the branch’s surface area) as shown in Figure 2B, a consistent monotonic decrease in particle density is apparent with each deeper generation as anticipated in the deep TB lung regions (Zhang et al., 2009). Namely, average particle density is observed to decrease by nearly half between the first and third generation, with a mean of 3.9 particles/mm^2^ in generation 1 (G1) compared to 2.2 particles/mm^2^ in generation 3 (G3). With our current design and exposure assay, we were able to reproduce spatial particle deposition density gradients across the airway trees and mimic anticipated *in situ* situations. Demonstrating this ability in a proof-of-concept holds ramifications for future cytotoxicity as well as drug screening assays (e.g., inhalation therapy), where local deposition patterns and thereby concentrations hotspots (Imai et al., 2012; Hofemeier and Sznitman, 2015; Islam et al., 2017), can lead to various differences in local inflammatory responses and disease onset. Such feature lies beyond reach using traditional assays (e.g., Transwell) or conversely straight channels.

To date, direct *in vivo* exposure studies of aerosol deposition have focused mainly on olfactory and upper trachea–bronchial regions in both animals (Gerde et al., 1991; Petitot et al., 2013) and humans (Cheng et al., 1996), with little explorations in the small bronchi and bronchioles. In addition, since *in vivo* data are conducted mostly with 2D gamma scintigraphy, the resolution of current imaging modalities remains inadequate to quantify deposition at small scales (Koullapis P. et al., 2018). Such limitations have driven the appeal of *in vitro–in silico* based strategies to deliver insight into deposition determinants. To date, however, efforts have been focused mainly on upper airways (Byron et al., 2010; Carrigy et al., 2014) with few studies exploring deposition endpoints in the mid- to small-bronchi. For example, Fishler et al. (2015) characterized the deposition of PS particles (i.e., 0.2–1 μm in diameter) in an *acinus-on-chip* platform and correlated findings *in silico*. In the footsteps of such endeavors, computational fluid dynamics (CFD) simulations were presently sought to gain quantitative insight into the deposition mechanisms at play. Results exhibit strong consistency when compared with the experimentally obtained deposition patterns (Figures 2C,D), with a maximum difference of 8% between experimental and numerical results. Notably, the deposition of 2 μm particles in small bronchi is principally governed by sedimentation, in line with the general understanding of inhalation aerosol deposition theory (Zhang et al., 2009). Dimensional analysis (see Supplementary Material) supports that deposition patterns under a horizontal orientation with respect to gravity are the result of the coupling between convective flow (i.e., viscous drag) along the streamwise airway direction and gravitational sedimentation in the normal direction, thereby giving rise to gradients in deposition density along each generation.

In addition, our *in vitro* exposure platform allows to examine the effect of gravity on ensuing deposition patterns by changing the orientation of the *airway-on-chips* (see Supplementary Material). Briefly, models were attached at a 45° tilt with respect to gravity and were exposed for 30 min to aerosolized 2 μm particles under physiological inhalation airflows. A decrease in the total number of deposited particles on the bottom PET membrane was observed as well as higher particle density in branches oriented lower with respect to gravity (see Supplementary Figure S3). Note that due to the imaged plane of the device (i.e., PET membrane), only deposited particles are identified (compared with those deposited on the PDMS side walls). Altogether, our *in vitro* exposure assays capture realistic aerosol transport determinants that still remain widely absent in more traditional PM instillations or direct spraying techniques (Blank et al., 2006; Lenz et al., 2014; Röhm et al., 2017) on plates and inserts. Furthermore, our *airway-on-chips* highlight for the first time that exposure assays can be configured to explore localized deposition effects, capturing for example deposition outcomes pertinent to regional lung lobes (e.g., upper versus lower lobes) that span broad orientation angles across the chest cavity (Sauret et al., 2002).

### Reconstituting a Bronchial Epithelium on Chip

**FIGURE 3:**
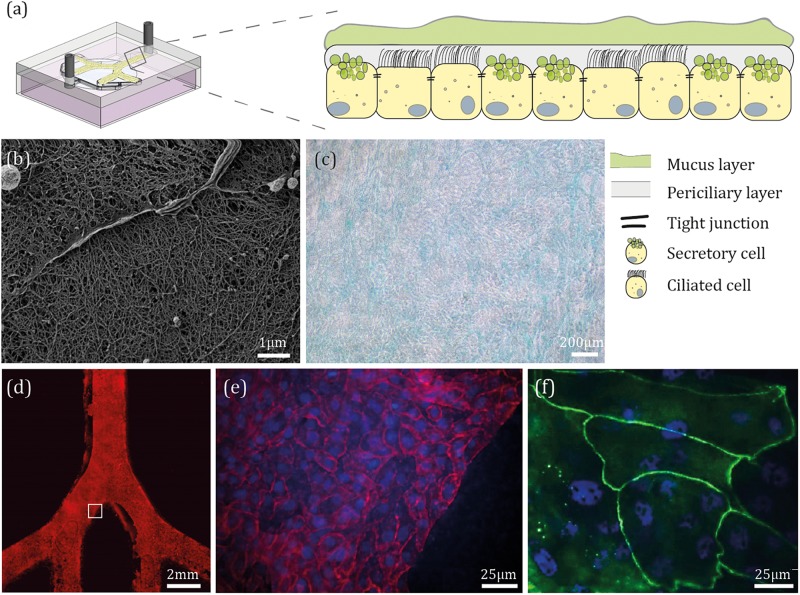
Epithelial barrier reconstitution inside *airway-on-chips*. **(a)** Schematic illustration of the bronchial epithelium environment and the general differentiated cellular make-up. **(b)** Following device fabrication, NHBE cells were seeded on the apical side of the PET membrane under immersed conditions for 4 days, then cultured at ALI conditions for 21 days. Shown here is a scanning electron microscope (SEM) image of the mucus layer covering NHBE cells. **(c)** After 21 days at ALI conditions, NHBE cells were stained for glycoproteins typically present in mucus. **(d)** NHBE cells were cultured for 7 days under immersed conditions, then stained for F-actin. **(e,f)** After 21 days at ALI conditions, NHBE cells were stained for F-actin (red), ZO1 (green), and cell nuclei (blue).

To reconstitute an epithelial cell population lining the conducting airways (e.g., ciliated and secretory cells), primary NHBE cells originating from healthy human bronchi (biopsy) were seeded on the apical side of the ALI device (Figure 3a). Although the use of primary cells is limited by difficulties in sample isolation, the small number of cells that can be produced and the large variation between different donors (Skardal et al., 2016), such strategy represents a well-acknowledged *in vitro* approach in mimicking generic airway models (Rayner et al., 2019) that capture the *in vivo* environment; a point advocated in recent *airway-on-chip* designs (Benam et al., 2015). By using NHBE cells, we have thus followed a similar approach, including the recent work of Bovard et al. (2018) who developed a *lung*/*liver-on-a-chip* platform with NHBE cells to study the toxicity of inhaled aerosols. Moreover, many studies have used such cells as an airway model to study cell exposure to ambient air pollution (Becker et al., 2005), using traditional assays with Transwell inserts. Among other, Wu et al. (2001) studied the expression of IL-8 through the activation of the EGF receptor signaling after NHBE cells were exposed to environmental PM.

To evaluate differentiation, following 21 days at ALI conditions, we visualized mucus secretion from the differentiated cells using SEM as shown in Figure 3b, where mucus microstructure is similar to that observed in previous lungs mucus studies (Schuster et al., 2013). Since washing off the mucus layer from the epithelial culture could potentially damage the differentiated NHBE cells (i.e., ciliated and secretory cells), we have reverted to SEM imaging of the epithelium layer that was seeded on top of an insert and cultured at an ALI for 21 days (Supplementary Figure S4a), in parallel to those within the devices and under the same growth conditions. As a control, the undifferentiated NHBE cells were also investigated using SEM imaging (Supplementary Figures S4c,d) to emphasize the fundamental changes in morphology. In addition, we visualized specific glycoproteins, present in mucus (Figure 3c; see Supplementary Figure S4f for staining control). A confluent cell monolayer was observed by staining the whole device for F-actin, as shown in Figure 3d, and cell nuclei (Figure 3e represents an inset of the white square in Figure 3d). Finally, to assess the barrier formation in the chip, expression of one of the tight junction proteins (i.e., ZO1) was examined (Figure 3f). In parallel, for control, each of the cells passages was seeded on an insert under the same growth conditions (see for example Supplementary Figure S4).

### Cell Viability Assay Following PM-Like Aerosol Exposure

Next, we explored the influence of PM-like particles on the NHBE cells cultured inside the device for cytotoxicity evaluation. To mimic the toxic characteristics of a PM group [e.g., oil fly ash (Agopyan et al., 2004), soils dust (Veranth et al., 2006), and coal fly ash (Deering-Rice et al., 2016) among others], we chose to simulate a well-known ligand that activates the Vanilloid receptor by preparing PM-like particles consisting of a PS-core conjugated to αVR1 antibodies (see the section “Particle Preparation”). We note that this activation can induce diverse biological cascades, including the release of pro-inflammatory cytokines such as IL-6, IL-8, and TNFα (Veronesi et al., 1999). Here, in a proof-of-concept, we specifically chose to demonstrate one well-known apoptosis pathway in the footsteps of previous studies (Agopyan et al., 2003, 2004).

**FIGURE 4:**
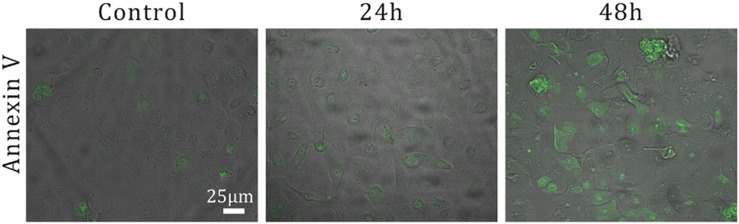
Examination of αVR1 antibody effect on NHBE cells over time. The apoptotic response over time for αVR1 antibody on NHBE cells was investigated as a positive control. 5 × 10^4^ NHBE cells per well are seeded in a 96-well plate. Two days after seeding, medium containing 0.66 μM αVR1 antibody was added, followed by incubation of 24 and 48 h. Next, apoptotic cells were stained using Annexin V (see the section “Apoptosis Quantification”), and imaging of fluorescent immunostaining was performed.

The conjugation of the αVR1 antibody to the PS particles (i.e., PS-VR1) was confirmed by incubating the PS-VR1 with a secondary antibody (Supplementary Figure S2). As previously shown *in vitro*, the binding of the αVR1 antibody to the VR1 receptor, which is expressed on the epithelial surface, induces an apoptosis pathway (Agopyan et al., 2003, 2004). Thus, a qualitative measurement of the apoptotic response as a function of time of the NHBE cells after a αVR1 antibody stimulation was first performed using an Annexin V staining which stains for cells that enter the apoptotic pathway. Following an incubation period of 48 h, an increase in the apoptotic cell population was observed (Figure 4). As a positive control, to demonstrate the influence of our PM-like particles on the NHBE cells, cells were incubated with PS-BSA and PS-VR1, in a particle:cell ratio of 1:50, for an incubation period of 48 h. A qualitative measurement of the apoptotic cells was performed using Annexin V staining (Supplementary Figure S5) showing an increase in apoptotic cells which were incubated with the PS-VR1 particles, compared to control (incubation with PS-BSA particles). In addition, the PS toxicity level was assessed by titration of the PS-BSA particles which were incubated for 48 h with NHBE cells in a range of particles:cells ratios (Supplementary Figure S6a). A decrease in cell viability was detected starting from a particles:cells ratio of 5:1 (the selected ratio is ∼1:500). The PS-VR1 toxicity effect was evaluated via viability assay following an incubation period of 48 h with NHBE cells in a particles:cells ratio of 1:100, resulting in a decrease in viability (Supplementary Figure S6b). This comprehensive examination allows us to refer to the manufactured PS-VR1 as PM-like particles, in line with seminal *in vitro* studies using traditional macroscopic assays on plate (Agopyan et al., 2003, 2004).

**FIGURE 5:**
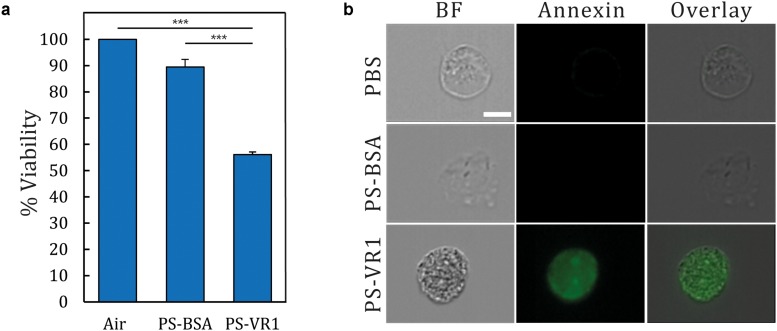
Viability and apoptosis assay following NHBE cell exposure to PM-like particles. **(a)** Following inhalation exposure assays, *airway-on-chip* devices were returned to the incubator for 48 h prior to conducting a viability assay. The presented results are normalized to the air exposure, representing 100% viability (*n* = 4 for each treatment). **(b)** Single cell imaging using Annexin V staining which stained apoptotic cells with green (BF corresponds to bright field). Error bar represent the standard error and asterisks indicate significance (*p* < 0.001 shown as ***).

Our developed *in situ-*like airway exposure platform is suitable for diverse applications in the field of human inhalation toxicity *in vitro*. As a proof-of-concept, our *airway-on-chips* were exposed to harmful PM-like particles and their effect on the human bronchial epithelium was monitored. Following previous studies (Agopyan et al., 2003, 2004), cells were grown under immersed conditions and only exposed to air during the specific exposure assay. Prior to the exposure assay, cells were grown inside the *airway-on-chips* until confluency was reached (5–8 days following seeding). The models were then taken out of the incubator and connected to the larger conducting 3D airway model (Figure 1c) where a 5 min inhalation exposure was performed inside the biological hood (at RT). Next, the devices were placed back at the incubator for 48 h incubation (see the section “Materials and Methods”). Experiments were carried out for three independent exposure protocols: air, PS-BSA, and PS-VR1, respectively, using four replicates. Viability measurements were then performed 48 h post-exposure, as shown in Figure 5A. The final percentage viability was normalized to the averaged value of the measurements for the air exposed group (defined as 100% viability). Our results indicate that the PS-BSA treatment was not toxic to the cells with around 90% viability. In contrast to the PS-BSA particles, the exposure to PS-VR1 particles led to a stark reduction in cell viability down to around 50% with standard error of 1.04%, demonstrating the high potency of PS-VR1 as an apoptosis inducer.

As the viability assay measures the level of the cellular metabolite and is not directly correlated with the level of apoptotic cells, a complementary assay was furthermore performed using Annexin V staining. The complete monolayer of cultured NHBE cells was trypsinized and harvested from the model, and was then incubated with Annexin V staining, followed by single cell imaging (see the section “Apoptosis Quantification”) for each group. Here we present in Figure 5B a characteristic image for each treatment illustrating the ensuing apoptotic levels; in the bright field (BF) column, single cells exhibiting a circular shape under each treatment show a typical morphology. When staining the cell membrane with Annexin V, only the cells under the PM-like treatment (i.e., PS-VR1) are stained compared with the controls (i.e., air and PS-BSA). This latter procedure further underlines one of the advantages of the present *airway-on-chip* platform; namely the ability to harvest cultured cells post exposure and conduct advanced single cell analyses. Together, these findings reinforce our setup as the first *in situ*-like platform suitable for aerosol exposure assays that combine realistic aerosol transport pathways in the lungs (i.e., from mouth to lumen) with biological toxicity that corroborates biological endpoints discussed in previous studies (Agopyan et al., 2003, 2004).

## Conclusion

While past efforts with *lung-on-chips* have been widely restricted to simple isolated airway channels, in the present work we have established an advanced *in vitro* platform to recapitulate *in situ*-like aerosol exposure to PM under ALI conditions. Multi-generational *airway-on-chip* devices, mimicking bifurcating airway structures at true scale, were exposed to inhaled aerosols under physiological airflows within an integrated anatomically inspired bronchial airway tree model. Using *in vitro–in silico* strategies, our experimental efforts underscore the importance of the small airway tree anatomy and its orientation to gravity in determining mechanistically driven lung deposition outcomes; an important step toward *in vitro* pathways to explore deposition outcomes for various real lung-like deposition scenarios. Such efforts are anticipated to help shed light on inhaled particle deposition in deep airways and mimic more realistically representative *in vivo* deposition outcomes in human lungs with heterogeneous patterns.

Furthermore, we demonstrated the aptitude to culture human primary cells under ALI conditions for 21 days, guaranteeing them to differentiate to ciliated and secretory cells and thereby deliver a functional *in vivo-*like barrier. Such design allows additionally to incorporate in future studies the presence for example of immune cells as well as other co-/multi-cell cultures for specific endpoints. As a proof-of-concept, we manufactured monodisperse particles that mimic PM-induced apoptosis through the activation of the Vanilloid receptor and used two complementary techniques to demonstrate the effect of such harmful PM-like particles on the human bronchial epithelium (i.e., a viability assay and imaging ensuing apoptotic levels). At this stage, our aim was foremost to demonstrate how PS-VR1-conjugated particles deposited under physiological airflow conditions lead to loss of viability of the epithelium via an apoptotic cascade. In future studies, our devices can lend use for example toward physiological-based particle size screens. Future directions include also quantifying the effects of occupational environments on the epithelium barrier as our platform can be leveraged for more general *in vitro* exposure with the potential to expand our current knowledge on the mechanisms in which the PM injure the bronchial epithelium.

## Data Availability Statement

All datasets generated for this study are included in the article/Supplementary Material.

## Author Contributions

SE-K conceived the project and devised the experiments, designed the device, performed the experiments, analyzed the data, and wrote the manuscript. AA-S designed the experiments, performed the experiments, and wrote the manuscript. PD and MH-A performed the numerical simulations. NK assisted with manuscript drafting. JS conceived the project, supervised the analyses, and wrote the manuscript.

## Conflict of Interest

The authors declare that the research was conducted in the absence of any commercial or financial relationships that could be construed as a potential conflict of interest.
