# Supplementary material for: In situ-Like Aerosol Inhalation Exposure for Cytotoxicity Assessment Using Airway-on-Chips Platforms
Source: Front Bioeng Biotechnol. 2020 Feb 20;8:91. doi: 10.3389/fbioe.2020.00091 (PMC7044134; doi:10.3389/fbioe.2020.00091)
Supplement: Supplementary file 1 [file Table_1.docx]

Supplementary Material: *In situ-*like aerosol inhalation exposure for cytotoxicity assessment using *airway-on-chips* platforms

Shani Elias-Kirma^1^, Arbel Artzy-Schnirman^1^, Prashant Das^1^, Metar Heller-Algazi^1^, Netanel Korin^1^ and Josué Sznitman^1*^

^1^ Department of Biomedical Engineering, Technion - Israel Institute of Technology, Haifa, Israel

*** Correspondence:**Josué Sznitman
[sznitman@bm.technion.ac.il](mailto:sznitman@bm.technion.ac.il)

**Table of Contents:**

Figure S1: Custom-designed 3D airway connector for *airway-on-chip* exposure assays.

Figure S2: Qualitative assessment of producing PM-like particles.

Figure S3: Aerosol deposition assay following exposure at 45° inclination.

Figure S4: Epithelial barrier reconstitution on a Transwell insert.

Figure S5: Examination of PM-like particle toxicity on NHBE cells.

Figure S6: Viability and apoptosis assays following NHBE cell exposure to PM-like particles in a 96 well plate.


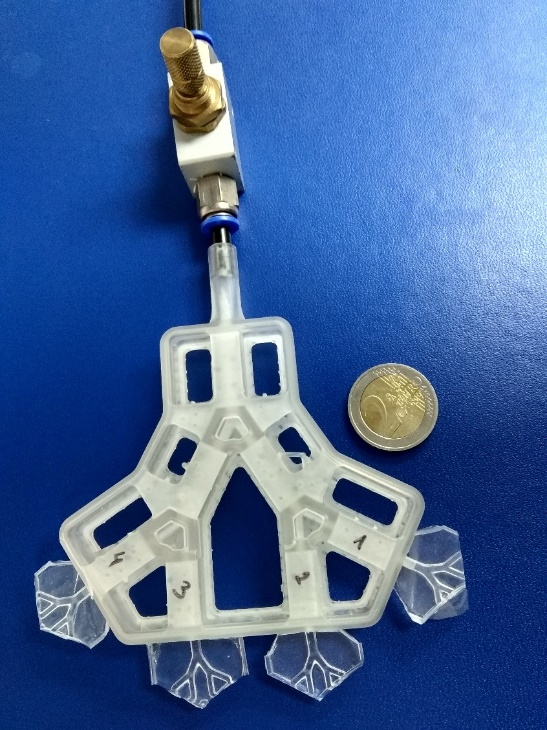


**Figure S1 Custom-designed 3D airway connector for *airway-on-chip* exposure assays.** Custom-designed 3D printed airway tree connector. The planar tree consists of three generations representative of mid-bronchial generations in an average adult human lung. The aerosol-laden airstream (see Methods) is fed through the inlet of the first generation of the connector. With such setup, the gravitational orientation of chips may be changed for a specific assay (see Results & Discussion). The *in vitro* setup allows to simultaneously perform four exposure assays, whereby *airway-on-chips* are directly connected to the four outlets of the connector (as shown in image).


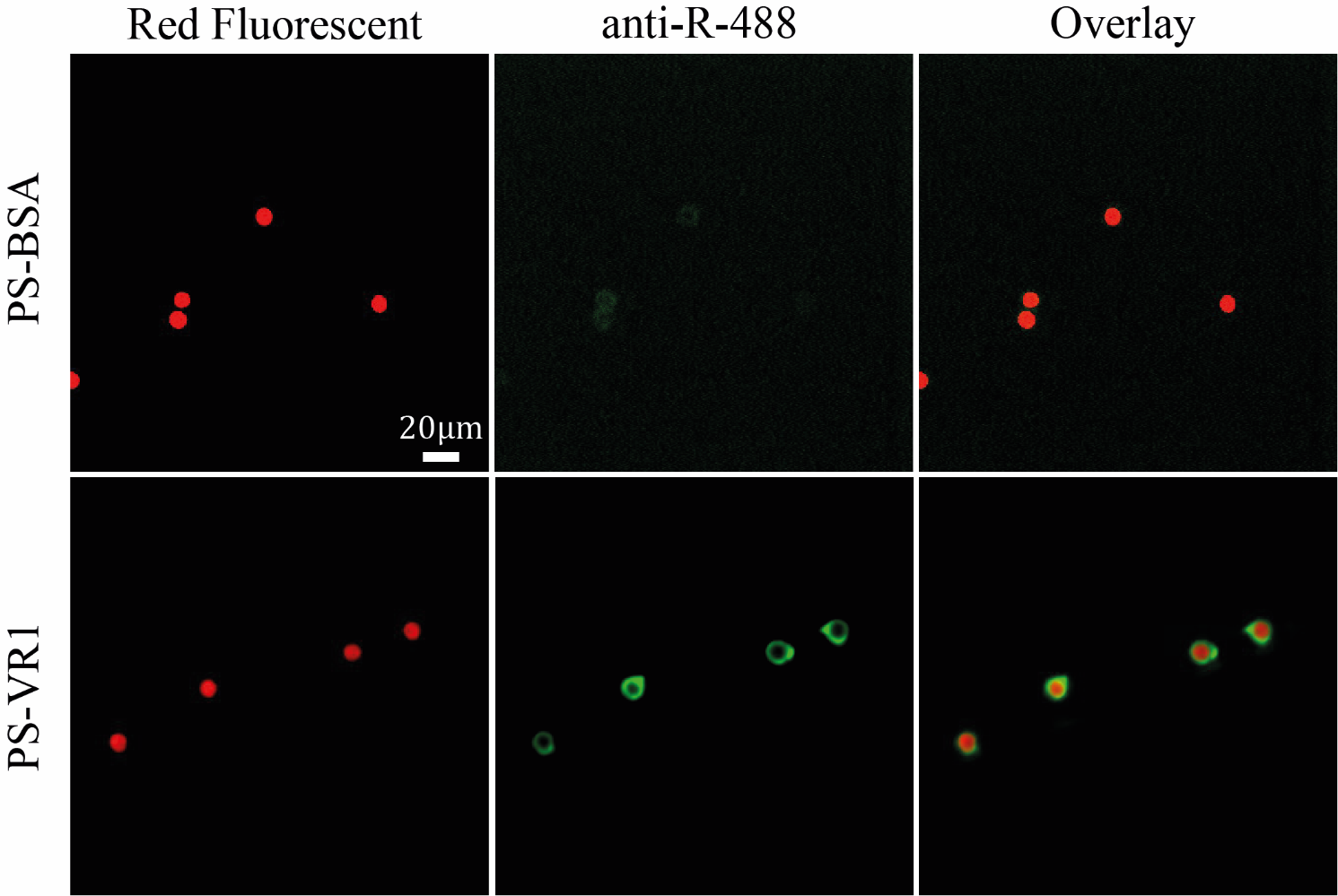


**Figure S2** **Qualitative assessment of producing PM-like particles.** To assess qualitatively the PS-VR1 conjugation, fluorescent PS particles (red) were incubated with secondary antibody Alexa Fluor 488 anti-rabbit for 1h at RT. Confocal microscopy imaging of fluorescent immunostaining is presented, emphasizing that αVR1 antibodies (labelled with green by the secondary antibody) were successfully conjugated to the PS particles.


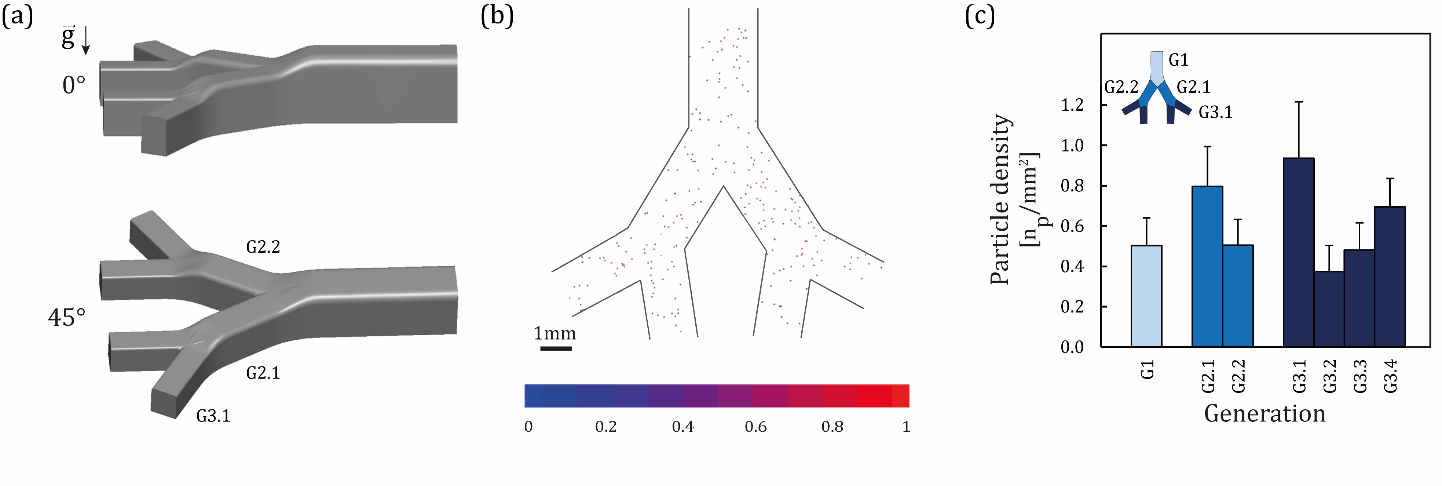


**Figure S3 Aerosol deposition assay following exposure at 45° inclination.** 2 µm PS particles were aerosolized and streamed into the model positioned at 45° with respect to gravity under physiological flow rate conditions (see Methods). (a) CAD illustration of the model’s position relative to gravity. (b) Ensemble deposition patterns for n=8 models imaged under fluorescence microscopy. The color-coded heat map quantifies particle concentration, defined as the number of neighbouring particles within a 0.5 mm radius (results are normalized by the highest concentration in the tree). (c) Histogram of particle deposition density quantifying the average particle number in each generation normalized by airway area (mm^2^). Error bars represent the standard error.

**Non-dimensional particle deposition analysis**

To better understand the mechanisms leading to deposition across the *airway-on-chips*, we analysed the characteristic non-dimensional Stokes (Stk), Péclet (Pe) and Gravity (Ga) numbers at each generation of the model, summarized in Table S1. Here, Stk = *ut_0_/d* represents the relative magnitude of the characteristic relaxation time of a particle, *t_0_* = *ρ_p_d_p_*/18*μ*, to the characteristic time of the flow (i.e. *u* is the mean airflow velocity in each generation of diameter *d*, *ρ_p_* is the particle density, *d_p_* the particle diameter, and *μ* is the dynamic viscosity of air). A low Stk number (i.e. Stk<<1) indicates that the 2 µm particles act as flow tracers, allowing us to neglect inertial effects and thus impaction as a deposition mechanism. Pe = *du*/*D* represents the relative magnitude of the advective transport rate to the diffusive transport rate (i.e. *d* is the channel height, *D* is the diffusion coefficient of the particle in air calculated using the Stokes–Einstein equation (Spielman, 1977)). The low 1/Pe number indicates the weak role of diffusive transport relative to convective transport. In addition, when comparing 1/Pe to Ga = *t_0_g/u* (i.e. the ratio of terminal settling velocity to the characteristic mean airflow velocity *u* in each generation), we find that 1/Pe is 3 order of magnitude larger than Ga, thereby indicating that particle deposition is mainly governed by gravity in our models.

**Table S1** Non-dimensional particle numbers in the *airway-on-chips*.

| Gen’ | Stk | 1/Pe | Ga |
| --- | --- | --- | --- |
| 1 | 1.0·10^-3^ | 3.1·10^-8^ | 7.4·10^-4^ |
| 2 | 1.1·10^-3^ | 4.8·10^-8^ | 8.6·10^-4^ |
| 3 | 1.4·10^-3^ | 7.1·10^-8^ | 9.5·10^-4^ |


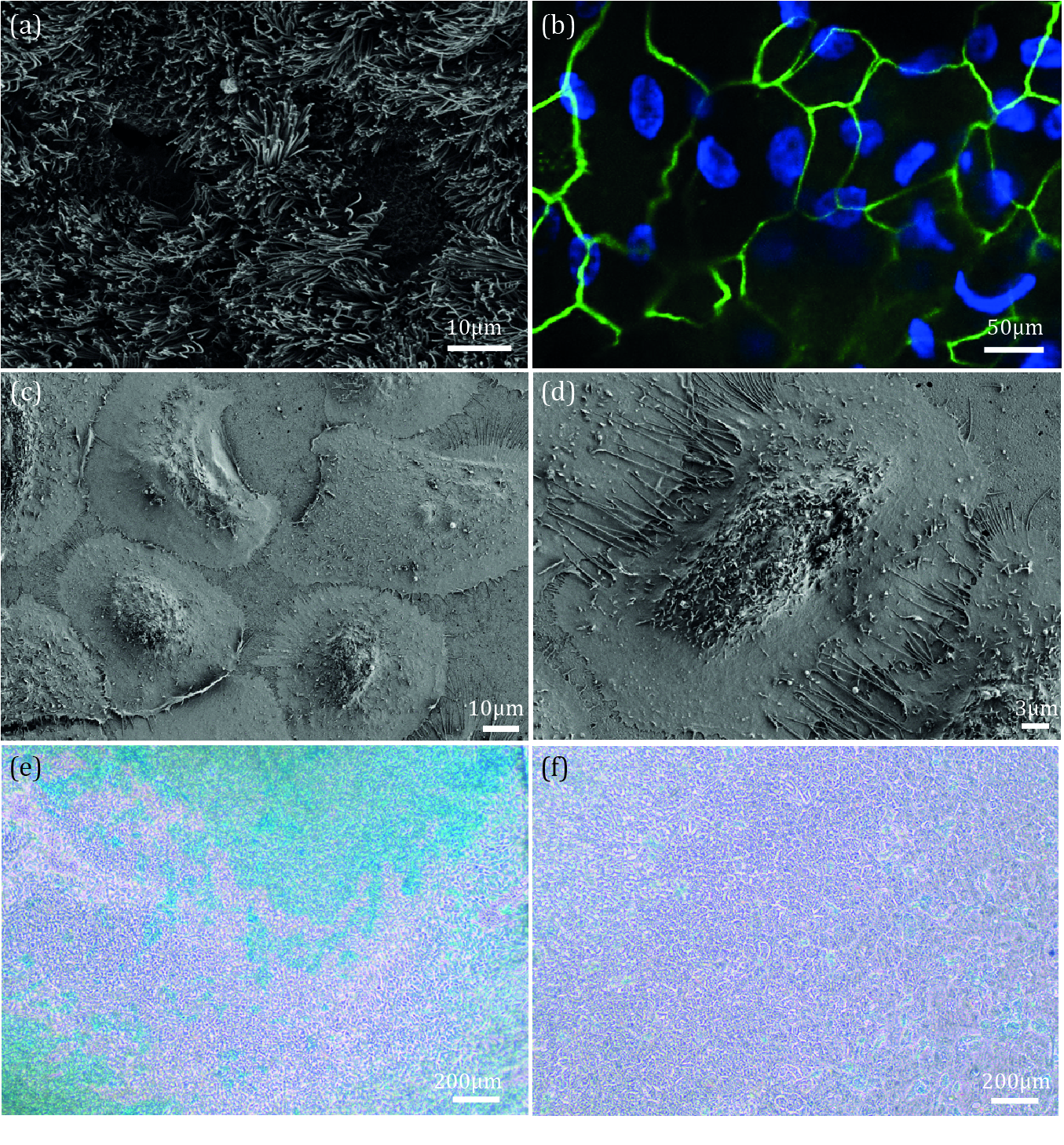


**Figure S4** **Epithelial barrier reconstitution on a Transwell insert.** NHBE cells were seeded on the apical side of a PET membrane insert under immersed conditions for 4 days, and then cultured at an air-liquid interface (ALI) for 21 days. (a) Scanning electron microscope (SEM) of the differentiated NHBE cells where ciliated and secretory cells can be observed. (b) NHBE cells were stained for ZO1 (green) and cell nuclei (blue) at the ALI. (c,d) As a control, the morphology of undifferentiated NHBE cells were investigated using SEM imaging. (e,f) To show explicit mucus staining, NHBE cells were stained for glycoproteins typically present in mucus and compared with human Alveolar Epithelial Lentivirus immortalized (hAELVi) cells (Artzy‐Schnirman et al., 2019) (which do not secret mucus).

**
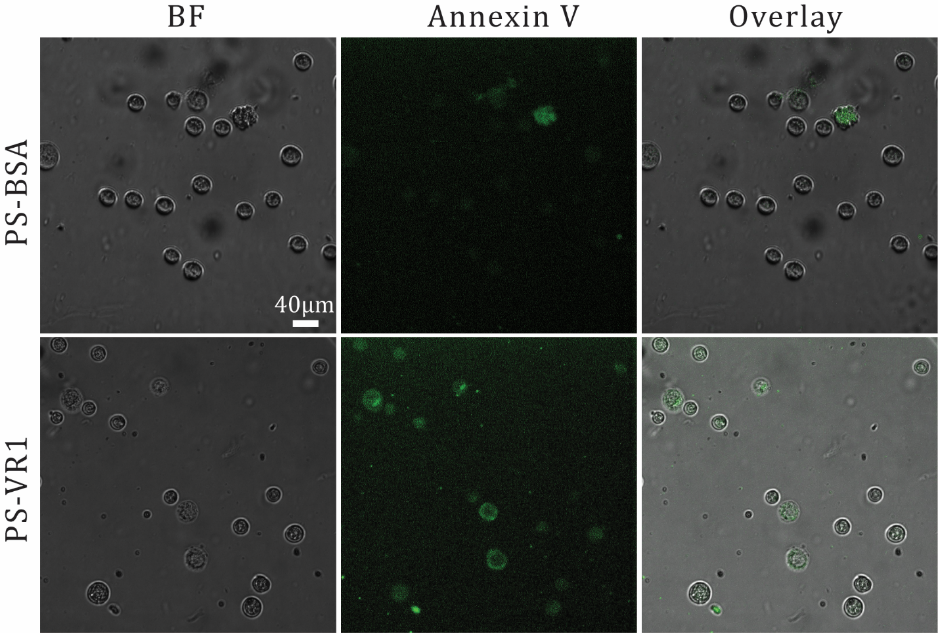
**

**Figure S5 Examination of PM-like particle toxicity on NHBE cells.** As a positive control, the propensity of 2 µm polystyrene (PS) particles conjugated with BSA (PS-BSA) and αVR1 antibody (PS-VR1) leading to apoptosis was assessed in NHBE cells. Briefly, 5x10^4^ NHBE cells were seeded on top of an insert for 7 days under immersed conditions, followed by 48h incubation with PS-BSA and PS-VR1, respectively, in a particle:cell ratio of 1:50. Next, cells were harvested and incubated with Annexin V (see Apoptosis quantification). Confocal imaging of fluorescent immunostaining is presented (BF corresponds to bright field imaging).


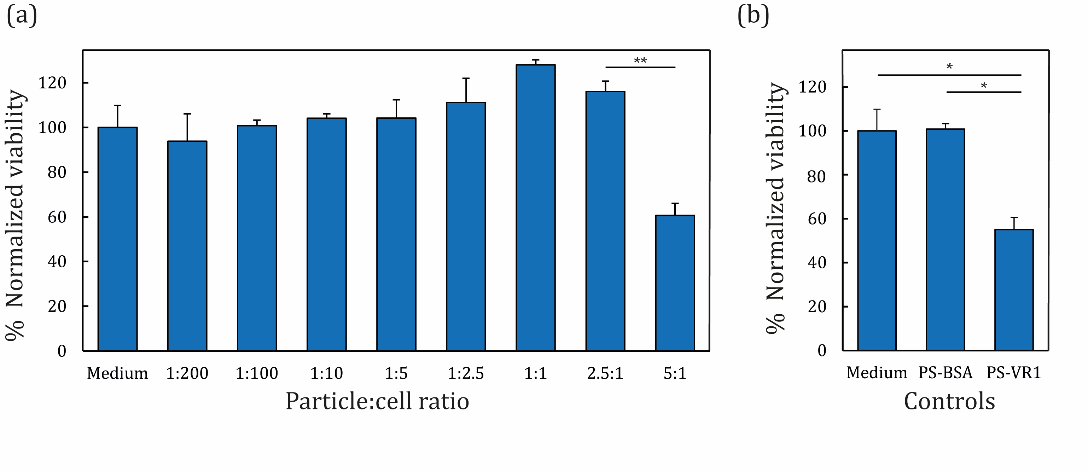


**Figure S6** **Viability and apoptosis assays following NHBE cell exposure to PM-like particles in a 96 well plate.** (a) Two days following seeding NHBE in a 96 well plate, medium containing 2 µm PS-BSA particles with a particle:cell ratios of 5:1, 2.5:1, 1:1, 1:2.5, 1:5, 1:10, 1:100 and 1:200, respectively, was added to each well, followed by 48h incubation. Next, a viability assay was performed using a cell viability reagent, i.e. almarBlue (n=3 for each treatment). (b) As a positive control, medium containing 2 µm PS-BSA and PS-VR1 particles with a particle:cell ratio of 1:100 was added in the same experiment and a viability assay using almarBlue was performed (n=3 for each treatment). All error bars presented correspond to standard errors (p<0.05, 0.005 shown as * and **, respectively).

# References

Artzy‐Schnirman, A., Zidan, H., Elias‐Kirma, S., Ben‐Porat, L., Tenenbaum‐Katan, J., Carius, P., et al. (2019). Capturing the Onset of Bacterial Pulmonary Infection in Acini‐On‐Chips. *Adv. Biosyst.* 1900026, 1900026. doi:10.1002/adbi.201900026.

Spielman, L. A. (1977). Particle Capture from Low-Speed Laminar Flows. *Annu. Rev. Fluid Mech.* 9, 297–319. doi:10.1146/annurev.fl.09.010177.001501.
